# Analysis of glycolytic flux as a rapid screen to identify low lactate producing CHO cell lines with desirable monoclonal antibody yield and glycan profile

**DOI:** 10.1186/1753-6561-5-S8-P94

**Published:** 2011-11-22

**Authors:** Rachel Legmann, Julie Melito, Ilana Belzer, David Ferrick

**Affiliations:** 1 Seahorse Bioscience, N. Billerica, MA, USA; 2 ProCognia, Ashdod, IL, USA

## Background

In CHO cell lines currently selected for the production of recombinant antibody, approximately 80% of the metabolized glucose is converted into lactic acid. These cells with a glycolytic phenotype exhibit significantly higher rates of proton production (extracellular acidification rate, ECAR) from lactate production than cells using oxidative phosphorylation (oxygen consumption rate, OCR). Therefore, shifts in the cell’s metabolism can be detected conveniently and dynamically through simultaneous detection of ECAR and OCR. Such measurements can characterize the metabolic programming of individual cell types and forecast the quality potential of their produced glycoproteins. In this study, we utilized an XF96 analyzer to measure glycolysis and mitochondrial respiration simultaneously, and in real-time. This allows one to determine the response of these two pathways to ATP demand, and indirectly, biosynthetic needs. A rapid screen was performed to determine the desired lactic acid production by exposing the cells to alternate sources of substrates, such as galactose or fructose. Specific metrics of the study included cell growth, product yield, and glycan profile. Higher titer, viable cell density, and viability along with glycol-similarity were observed for galactose and glutamine feeding strategies during the production phase. We believe this rapid, cell based metabolic screen that is label-free and non-invasive can be used to identify low lactic acid CHO mAb cell producers in both batch and fed-batch systems. This selection is accomplished without compromising clone productivity and product quality.

## Material and methods

**Figure 1:**
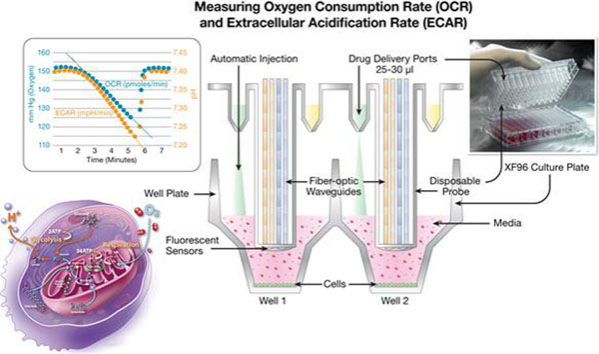
Seahorse XF analyzer measure the two ATP generating pathways of the cell. The cell is consuming oxygen, producing CO_2_ and excreting (H+) protons that acidify the media.

Suspended CHO cells producing a recombinant IgG monoclonal antibody (MAb) were maintained in high glucose serum free chemically-defined (CD) CHO supplemented with 0.2μM methotrexate (MTX). The low-buffered DMEM assay medium was used for XF96 ECAR screening and CD CHO medium was used for fed-batch flasks for lactic acid screening. Mitochondrial function and cellular bioenergetics were measured in intact CHO_mAb using a Seahorse Bioscience extracellular flux analyzer (XF96) as described previously (1, 3) and demonstrated in Figure 1. The sensor cartridge is embedded with 96 dual florescent biosensors (O and H+). Each sensor cartridge is also equipped delivery ports for injecting agents during an assay. Cells were maintained in a 5% CO2 incubator at 37 °C and 1 h before the experiment, cells were washed and incubated in nonbuffered (without sodium carbonate) DMEM (sugar-free) pH 7.4, at 37 °C in a non-CO2 incubator. Real time cellular ECAR and OCR were measured simultaneously before and after the substrate source injection. The metabolic rate of the cell population was measured repeatedly over about 30 min. Lactic acid concentrations in the flasks were measured after different carbon sources were added to the cells after 5 days of growth and glucose depletion during the production phase using a NOVA BioProfile 100 Plus. MAb concentrations in samples from flasks were quantified using Octet^QK^ instrument with protein A biosensors. Glycan profile in crude harvest samples from flasks were quantified using lectin based array by Procognia Glycoscope instrument described previously(2).

The illustration demonstrates how the XF96 measures changes in the microenvironment (3μl) surrounding live cells in a 96-well microplate. The O2 and proton concentration is determined by a quenching reaction of a fluorophore specific for O2 (blue) or protons (red) that is embedded into a single polymer martix on the biosensor cartridge.

## Results

**Figure 2:**
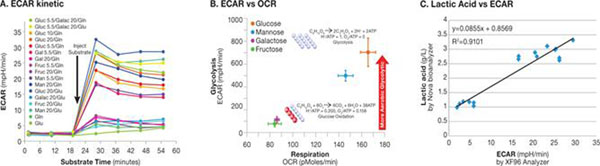
The effect of different carbon sources on ECAR from lactic acid production and on metabolic shift in *CHO_mAb.* Change of baseline ECAR of CHO_mAb cells after Carbon/nitrogen source injection versus time (A). Glucose and mannose preference for aerobic glycolysis(B) Comparison of maximum ECAR in XF96 with lactic acid in shake flasks showed an excellent correlation, R^2^=0.91 (C)

Mammalian cells in culture have inefficient glucose metabolism where most of the glucose consumed is converted into lactate, while very little is oxidized in the tricarboxylic acid cycle (TCA).One of the interpretation for this phenomenon of high glycolysis rate even under fully aerobic condition is that lactate generation from pyruvate may be used by these cells as a way to re-equilibrate their redox potential. The high glycolysis rates would generate NADH at high rates, which should be reduced to NAD in order to keep this pathway functional. In Figure 2(A), metabolic analyses from this study demonstrate that different carbon and nitrogen sources added during the product production phase can promote or diminish aerobic glycolysis as indicated by ECAR. The data show that ECAR correlates well with glycolysis driving lactic acid synthesis in the recombinant CHO_mAb cells. The highest lactic acid concentration and ECAR were obtained when glucose was present in the medium as the sole carbon source. The lowest lactic acid and ECAR were obtained when galacose or fructose was present in the media during the glycoprotein production phase. Therefore, adding galactose or fructose when glucose becomes depleted at the end of the growth phase would provide an alternative carbon source to drive low-waste lactic acid production. The rapid method developed in this study identified the optimum carbon and nitrogen source that achieved less than 77% proton production compared to glucose while maintaining optimal and consistent protein glycosylation. Metabolic shift to aerobic glycolysis was observed when glucose or mannose was injected into the CHO_mAb cell cultures as shown in Figure 2B. XF analyses demonstrated that cells consuming glucose or mannose are diverted toward glycolysis even in the presence of sufficient levels of dissolved oxygen. Good correlation was observed between ECAR and accumulation of lactic acid in Erlenmeyer flasks (Figure 2C).

## Conclusions

In this study, we developed and validated a rapid assay, employing the XF96 analyzer, to screen for low ECAR producing cells to predict low lactic acid production. The experiments show that there is a good agreement between ECAR and lactic acid and therefore ECAR can be rapidly measured as an index of glycolytic activity and serve as an accurate surrogate of lactate production. The data show that alternating carbon sugars during the production phase ,such as galactose and fructose, can help to control glycolysis and, therefore, reduce the lactate accumulation in mammalian cell cultures without compromising on recombinant glycoprotein productivity and desired glycoform profile.
